# Di-μ-hydroxido-bis[tris(1,1,1,5,5,5-hexa­fluoro­acetyl­acetonato-κ^2^ *O*,*O*′)hafnium(IV)] acetone solvate

**DOI:** 10.1107/S1600536809041658

**Published:** 2009-10-17

**Authors:** J. Augustinus Viljoen, Hendrik G. Visser, Andreas Roodt, Maryke Steyn

**Affiliations:** a Department of Chemistry, University of the Free State, PO Box 339, Bloemfontein, 9300, South Africa

## Abstract

The binuclear title compound, [Hf_2_(C_5_HF_6_O_2_)_6_(OH)_2_]·C_3_H_6_O, contains an Hf^IV^ atom which is eight coordinated and surrounded by three chelating β-diketonato 1,1,1,5,5,5-hexa­fluoro­acetyl­acetonate (hfaa) ligands and two bridging OH groups situated on a twofold rotation axis. The HfO_8_ coordination polyhedron shows a slightly distorted Archimedean square anti-prismatic coordination with average Hf—O, C—O, C—C_Me_ distances of 2.19 (2), 1.26 (2) and 1.49 (2) Å, respectively, and an O—Hf—O bite angle of 75.3 (5)°. Weak O—H⋯O hydrogen bonding inter­actions are observed between one of the bridging hydr­oxy groups and the disordered solvent mol­ecule.

## Related literature

A monoclinic structure of the solvent-free title compound was first investigated by Zherikova *et al.* (2006*a* ▶). For more hafnium and zirconium complexes containing β-diketonato ligands, see: Viljoen *et al.* (2008 ▶); Calderazzo *et al.* (1998 ▶); Zherikova *et al.* (2005 ▶, 2006*b* ▶); Steyn *et al.* (2008 ▶).
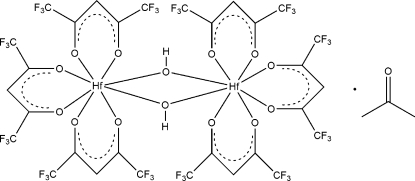


## Experimental

### 

#### Crystal data

[Hf_2_(C_5_HF_6_O_2_)_6_(OH)_2_]·C_3_H_6_O
*M* *_r_* = 1691.42
Monoclinic, 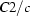

*a* = 22.1290 (14) Å
*b* = 12.4100 (8) Å
*c* = 19.5010 (11) Å
β = 105.197 (2)°
*V* = 5168.1 (6) Å^3^
*Z* = 4
Mo *K*α radiation
μ = 4.21 mm^−1^
*T* = 100 K
0.26 × 0.21 × 0.02 mm

#### Data collection

Bruker X8 APEXII 4K Kappa CCD diffractometer
Absorption correction: multi-scan (*SADABS*; Bruker, 2004 ▶) *T*_min_ = 0.360, *T*_max_ = 0.919
24620 measured reflections
6381 independent reflections
5035 reflections with *I* > 2σ(*I*)
*R*_int_ = 0.050

#### Refinement

*R*[*F* ^2^ > 2σ(*F* ^2^)] = 0.041
*wR*(*F* ^2^) = 0.119
*S* = 1.03
6381 reflections
373 parameters
5 restraints
H atoms treated by a mixture of independent and constrained refinement
Δρ_max_ = 2.07 e Å^−3^
Δρ_min_ = −1.49 e Å^−3^

### 

Data collection: *APEX2* (Bruker, 2005 ▶); cell refinement: *SAINT-Plus* (Bruker, 2004 ▶); data reduction: *SAINT-Plus*; program(s) used to solve structure: *SIR92* (Altomare *et al.*, 1999 ▶); program(s) used to refine structure: *SHELXL97* (Sheldrick, 2008 ▶); molecular graphics: *DIAMOND* (Brandenburg & Putz, 2005 ▶); software used to prepare material for publication: *WinGX* (Farrugia, 1999 ▶).

## Supplementary Material

Crystal structure: contains datablocks global, I. DOI: 10.1107/S1600536809041658/wm2265sup1.cif

Structure factors: contains datablocks I. DOI: 10.1107/S1600536809041658/wm2265Isup2.hkl

Additional supplementary materials:  crystallographic information; 3D view; checkCIF report

## Figures and Tables

**Table:** 

Hf—O1	2.258 (4)
Hf—O2	2.147 (4)
Hf—O3	2.209 (4)
Hf—O4	2.150 (4)
Hf—O5	2.238 (4)
Hf—O6	2.137 (4)
Hf—O7	2.113 (3)
Hf—O8	2.096 (3)

**Table:** 

O2—Hf—O1	75.83 (14)
O4—Hf—O3	74.36 (16)
O6—Hf—O5	75.77 (14)
Hf—O7—Hf^i^	112.5 (3)
Hf^i^—O8—Hf	113.9 (3)

Symmetry code: (i) 
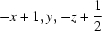
.

**Table 2:** Hydrogen-bond geometry (Å, °)

*D*—H⋯*A*	*D*—H	H⋯*A*	*D*⋯*A*	*D*—H⋯*A*
O7—H7⋯O01	0.81 (3)	1.98 (3)	2.783 (10)	170.0 (4)

**Fig. 1.**
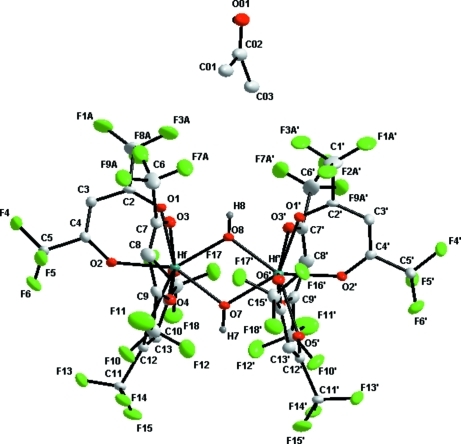
Representation of the title compound (I), showing the numbering scheme and displacement ellipsoids (50% probability). Only one orientation of the disordered parts of the structure (–CF3 and solvate) is displayed; hydrogen atoms are omitted for clarity.

**Fig. 2.**
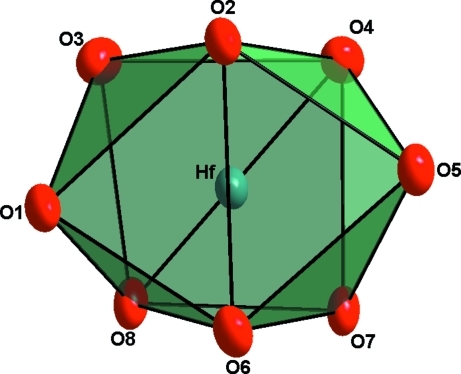
Slightly distorted Archimedean antiprism coordination polyhedron surrounding the central hafnium atom (displacement ellipsoids at the 50% probability level).

**Fig. 3.**
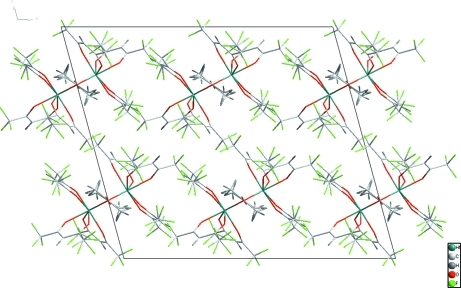
Packing of molecules in the crystal structure, illustrating how the dimer units are connected by Van der Waals interactions to form a three dimensional network (hydrogen atoms and F···F interactions omitted for clarity).

## supplementary crystallographic information

### Comment

This study was done as part of ongoing research into the reactions of *O,O'*- and *O,N*-bidentate ligands with hafnium(IV) and zirconium(IV). If hafnium and zirconium show differences in their chelating behaviour, either by reaction rates, solubilities, coordination modes, equilibrium behaviour, *etc*., it could possibly be exploited as a novel separation technique for the two metals. A wide range of volatile tetrakis-diketonato metal complexes have been prepared by Zherikova *et al*. (2005, 2006*a,b*) to be used for the preparation of hafnium dioxide films and oxide coatings.

Colourless crystals of the title compound crystallize in the monoclinic crystal system (*C*2/*c, Z*=4) (Figure 1) with four acetone solvent molecules in the unit cell. The monoclinic structure of the solvent-free compound earlier reported by Zherikova *et al. * (2006*a*) cannot be superimposed with the title compound due to differences in metal coordination. Hexafluoroacetylacetonato hafnium(IV) is one of very few complexes which is not isostructural to its zirconium counterpart. Literature revealed that Zr(hfaa)_4_ has a monomeric structure with a slightly distorted antiprismatic coordination polyhedron about the zirconium atom. The metal complex of the title compound consists of a Hf^IV^ atom which is eight-coordinated and surrounded by three hfaa ligands and two bridging OH-groups thereby adopting a slightly distorted Archimedean anti-prismatic coordination geometry (Figure 2). The dimer skeleton exhibits a flat diamond-like structure with Hf—O7, Hf—O8 and Hf—Hf^i^distances of 2.113 (7), 2.091 (7) and 3.5130 (7) Å, respectively, and a bite angle of 66.6 (4)°. The hexafluoroacetylacetonato ligands form three six-membered metallocycles with average Hf—O, C—O, C—C_Me_ distances of 2.19 (2) Å, 1.256 (15) Å and 1.485 (16) Å respectively, and an O—Hf—O bite angle of 75.3 (5)° (Table 1). In the title structure the dimer units are connected by Van der Waals interactions between different F atoms (Figure 3) to produce a three dimensional network, where the average F···F distances are 2.9 (2) Å. Lastly, weak hydrogen bonding interactions are observed between one of the bridging hydroxy groups (O7—H7) and the solvent molecule (Table 2). The opposing hydroxy group (O8—H8) does not show any hydrogen interactions, probably due to packing effects.

For more hafnium and zirconium complexes containing β-diketonato ligands, see Viljoen *et al.* (2008), Calderazzo *et al.* (1998), Zherikova *et al.* (2005, 2006b) and Steyn *et al.* (2008).

### Experimental

Chemicals were purchased from Sigma and Aldrich and used as received. Hexafluoroacetylacetone (450 µ*L*, 3.3 mmol) was added drop-wise to a suspension of HfCl_4_ (241 mg, 0.75 mmol) in toluene (10 ml). The dissolution turned into a slightly white solution after 20 min. After refluxing for *ca*. 12 h, the crude product was filtered and evaporated *via* vacuum. Colourless crystals were obtained after re-crystallization in acetone at 253 K. The compound crystallized out as a colourless substance. (Yield: 408 mg, 43%) Spectroscopy data: ^1^H NMR (acetone-*d_6_*): *δ* = 6.59 (s, H),7.29 (s, H); IR (ATR): *ν*(CO)= 1553 cm^-1^.

### Refinement

The aromatic, methine, and methyl H atoms were placed in geometrically idealized positions (C—H = 0.93–0.98) and constrained to ride on their parent atoms with *U*_iso_(H) = 1.2*U*_eq_(C) for aromatic and methine, and *U*_iso_(H) = 1.5*U*_eq_(C) for methyl protons. Torsion angles for methyl protons were refined from electron density. The highest residual electron density lies within 0.84 Å from F1B. Two of the CF_3_ groups are disordered over two positions each, and the anisotropic displacement parameters for these disordered groups were refined using similarity restraints. The acetone solvent molecule is disordered over two equal positions. The symmetry-related acetone is generated by the symmetry operator 1 - *x*, *y*, 1/2 - *z*, resulting in one full-occupancy disordered solvent.

### Crystal data

**Table:**

[Hf_2_(C_5_HF_6_O_2_)_6_(OH)_2_]·C_3_H_6_O	*F*(000) = 3200
*M**_r_* = 1691.42	*D*_x_ = 2.174 Mg m^−^^3^
Monoclinic, *C*2/*c*	Mo *K*α radiation, λ = 0.71069 Å
Hall symbol: -C 2yc	Cell parameters from 8999 reflections
*a* = 22.1290 (14) Å	θ = 3.1–28.1°
*b* = 12.4100 (8) Å	µ = 4.21 mm^−^^1^
*c* = 19.5010 (11) Å	*T* = 100 K
β = 105.197 (2)°	Plate, colourless
*V* = 5168.1 (6) Å^3^	0.26 × 0.21 × 0.02 mm
*Z* = 4	

### Data collection

**Table:**

Bruker X8 APEXII 4K Kappa CCD diffractometer	6381 independent reflections
Radiation source: fine-focus sealed tube	5035 reflections with *I* > 2σ(*I*)
graphite	*R*_int_ = 0.050
ω– and φ–scans	θ_max_ = 28.3°, θ_min_ = 1.9°
Absorption correction: multi-scan (*SADABS*; Bruker, 2004)	*h* = −25→29
*T*_min_ = 0.360, *T*_max_ = 0.919	*k* = −16→15
24620 measured reflections	*l* = −25→15

### Refinement

**Table:**

Refinement on *F*^2^	5 restraints
Least-squares matrix: full	H atoms treated by a mixture of independent and constrained refinement
*R*[*F*^2^ > 2σ(*F*^2^)] = 0.041	*w* = 1/[σ^2^(*F*_o_^2^) + (0.0606*P*)^2^ + 32.7892*P*] where *P* = (*F*_o_^2^ + 2*F*_c_^2^)/3
*wR*(*F*^2^) = 0.119	(Δ/σ)_max_ = 0.001
*S* = 1.03	Δρ_max_ = 2.07 e Å^−^^3^
6381 reflections	Δρ_min_ = −1.49 e Å^−^^3^
373 parameters	

### Special details

**Table:**

Geometry. All e.s.d.'s (except the e.s.d. in the dihedral angle between two l.s. planes) are estimated using the full covariance matrix. The cell e.s.d.'s are taken into account individually in the estimation of e.s.d.'s in distances, angles and torsion angles; correlations between e.s.d.'s in cell parameters are only used when they are defined by crystal symmetry. An approximate (isotropic) treatment of cell e.s.d.'s is used for estimating e.s.d.'s involving l.s. planes.
Refinement. Refinement of *F*^2^ against ALL reflections. The weighted *R*-factor *wR* and goodness of fit *S* are based on *F*^2^, conventional *R*-factors *R* are based on *F*, with *F* set to zero for negative *F*^2^. The threshold expression of *F*^2^ > 2σ(*F*^2^) is used only for calculating *R*-factors(gt) *etc*. and is not relevant to the choice of reflections for refinement. *R*-factors based on *F*^2^ are statistically about twice as large as those based on *F*, and *R*- factors based on ALL data will be even larger.

### Fractional atomic coordinates and isotropic or equivalent isotropic displacement parameters (Å^2^)

**Table:**

	*x*	*y*	*z*	*U*_iso_*/*U*_eq_	Occ. (<1)
Hf	0.419608 (10)	0.227714 (18)	0.207046 (12)	0.02116 (9)	
C1	0.3711 (5)	−0.0844 (7)	0.0809 (6)	0.0721 (14)	
C2	0.3592 (3)	0.0258 (4)	0.1079 (3)	0.0270 (12)	
C3	0.2981 (3)	0.0645 (5)	0.0884 (3)	0.0327 (13)	
H1	0.2669	0.0246	0.0577	0.039*	
C4	0.2838 (3)	0.1603 (5)	0.1140 (3)	0.0268 (12)	
C5	0.2168 (3)	0.2039 (5)	0.0939 (4)	0.0377 (16)	
C6	0.3336 (4)	−0.0077 (8)	0.3312 (5)	0.0638 (11)	
C7	0.3517 (3)	0.1044 (5)	0.3105 (4)	0.0356 (14)	
C8	0.3298 (3)	0.1955 (6)	0.3383 (4)	0.0386 (15)	
H2	0.2994	0.19	0.3633	0.046*	
C9	0.3545 (3)	0.2930 (5)	0.3274 (3)	0.0326 (14)	
C10	0.3404 (3)	0.3945 (6)	0.3660 (4)	0.0438 (17)	
C11	0.3635 (3)	0.5603 (5)	0.1098 (3)	0.0298 (13)	
C12	0.3980 (3)	0.4503 (5)	0.1192 (3)	0.0261 (12)	
C13	0.4324 (3)	0.4228 (5)	0.0712 (3)	0.0297 (12)	
H3	0.4415	0.4745	0.0409	0.036*	
C14	0.4526 (3)	0.3192 (5)	0.0693 (3)	0.0271 (12)	
C15	0.4830 (3)	0.2826 (5)	0.0116 (3)	0.0346 (15)	
O1	0.40552 (18)	0.0693 (3)	0.1480 (2)	0.0287 (9)	
O2	0.32118 (18)	0.2240 (3)	0.1555 (2)	0.0256 (8)	
O3	0.38704 (18)	0.1031 (3)	0.2699 (2)	0.0321 (9)	
O4	0.39107 (18)	0.3134 (3)	0.2892 (2)	0.0270 (8)	
O5	0.38857 (18)	0.3933 (3)	0.1676 (2)	0.0266 (8)	
O6	0.4472 (2)	0.2418 (3)	0.1103 (2)	0.0283 (9)	
O7	0.5	0.3223 (4)	0.25	0.0250 (11)	
O8	0.5	0.1356 (5)	0.25	0.0271 (12)	
F1A	0.3226 (8)	−0.1422 (12)	0.0495 (9)	0.0721 (14)	0.375 (6)
F2A	0.3984 (7)	−0.0611 (10)	0.0202 (8)	0.0721 (14)	0.375 (6)
F3A	0.4211 (8)	−0.1344 (12)	0.1156 (9)	0.0721 (14)	0.375 (6)
F1B	0.3629 (4)	−0.1582 (6)	0.1313 (5)	0.0721 (14)	0.625 (6)
F2B	0.3347 (5)	−0.1112 (7)	0.0202 (5)	0.0721 (14)	0.625 (6)
F3B	0.4280 (5)	−0.1047 (7)	0.0806 (5)	0.0721 (14)	0.625 (6)
F4	0.17688 (18)	0.1341 (4)	0.0530 (3)	0.0613 (13)	
F5	0.19641 (18)	0.2225 (3)	0.1501 (3)	0.0489 (11)	
F6	0.2136 (2)	0.2953 (4)	0.0574 (2)	0.0541 (12)	
F7A	0.3759 (4)	−0.0493 (8)	0.3801 (6)	0.0638 (11)	0.667 (12)
F8A	0.3265 (4)	−0.0786 (7)	0.2726 (5)	0.0638 (11)	0.667 (12)
F9A	0.2780 (4)	−0.0118 (7)	0.3431 (6)	0.0638 (11)	0.667 (12)
F7B	0.3875 (8)	−0.0651 (16)	0.3596 (11)	0.0638 (11)	0.333 (12)
F8B	0.3023 (8)	−0.0593 (14)	0.2764 (10)	0.0638 (11)	0.333 (12)
F9B	0.2993 (8)	−0.0006 (14)	0.3789 (12)	0.0638 (11)	0.333 (12)
F10	0.3100 (2)	0.4661 (4)	0.3203 (2)	0.0553 (12)	
F11	0.3059 (3)	0.3715 (5)	0.4103 (3)	0.0885 (19)	
F12	0.3928 (2)	0.4404 (4)	0.4033 (2)	0.0654 (14)	
F13	0.30844 (18)	0.5497 (3)	0.0616 (2)	0.0451 (10)	
F14	0.3532 (2)	0.5951 (3)	0.1687 (2)	0.0456 (10)	
F15	0.3962 (2)	0.6343 (3)	0.0856 (3)	0.0619 (13)	
F16	0.4473 (2)	0.2113 (4)	−0.0304 (2)	0.0474 (11)	
F17	0.53881 (19)	0.2378 (4)	0.0391 (2)	0.0482 (11)	
F18	0.4919 (2)	0.3644 (4)	−0.0295 (2)	0.0534 (11)	
H8	0.5	0.0703 (17)	0.25	0.064*	
H7	0.5	0.388 (2)	0.25	0.064*	
C02	0.5	0.6393 (9)	0.25	0.061 (2)	
O01	0.5047 (7)	0.5448 (7)	0.2346 (6)	0.061 (2)	0.5
C01	0.4627 (7)	0.6837 (13)	0.2974 (8)	0.061 (2)	0.5
H01A	0.4339	0.7369	0.272	0.091*	0.5
H01B	0.4904	0.7162	0.3384	0.091*	0.5
H01C	0.4398	0.6264	0.3122	0.091*	0.5
C03	0.5346 (8)	0.7260 (12)	0.2166 (9)	0.061 (2)	0.5
H03A	0.5757	0.7005	0.2174	0.091*	0.5
H03B	0.538	0.7915	0.2435	0.091*	0.5
H03C	0.5114	0.7393	0.1684	0.091*	0.5

### Atomic displacement parameters (Å^2^)

**Table:**

	*U*^11^	*U*^22^	*U*^33^	*U*^12^	*U*^13^	*U*^23^
Hf	0.02293 (13)	0.01426 (14)	0.02648 (14)	0.00092 (8)	0.00681 (9)	−0.00103 (9)
C1	0.085 (3)	0.037 (2)	0.088 (4)	0.018 (2)	0.013 (2)	−0.027 (2)
C2	0.033 (3)	0.011 (3)	0.037 (3)	0.001 (2)	0.010 (2)	−0.002 (2)
C3	0.029 (3)	0.025 (3)	0.038 (3)	0.000 (2)	−0.002 (2)	−0.006 (3)
C4	0.027 (3)	0.023 (3)	0.027 (3)	0.004 (2)	0.003 (2)	0.001 (2)
C5	0.026 (3)	0.025 (3)	0.054 (4)	0.001 (2)	−0.001 (3)	−0.009 (3)
C6	0.047 (2)	0.056 (2)	0.091 (3)	−0.0059 (17)	0.0231 (18)	0.031 (2)
C7	0.030 (3)	0.033 (4)	0.044 (4)	−0.003 (3)	0.009 (3)	0.011 (3)
C8	0.036 (3)	0.039 (4)	0.049 (4)	0.004 (3)	0.025 (3)	0.009 (3)
C9	0.032 (3)	0.036 (4)	0.028 (3)	0.009 (3)	0.005 (2)	0.002 (3)
C10	0.052 (4)	0.049 (5)	0.036 (4)	0.018 (3)	0.021 (3)	0.005 (3)
C11	0.034 (3)	0.015 (3)	0.037 (3)	0.003 (2)	0.002 (3)	−0.001 (2)
C12	0.025 (3)	0.020 (3)	0.030 (3)	0.002 (2)	0.003 (2)	0.000 (2)
C13	0.038 (3)	0.026 (3)	0.025 (3)	0.002 (2)	0.008 (2)	0.002 (2)
C14	0.026 (3)	0.029 (3)	0.025 (3)	−0.004 (2)	0.005 (2)	−0.002 (2)
C15	0.036 (3)	0.043 (4)	0.025 (3)	−0.002 (3)	0.010 (3)	−0.007 (3)
O1	0.026 (2)	0.017 (2)	0.042 (2)	−0.0005 (16)	0.0075 (17)	−0.0068 (18)
O2	0.024 (2)	0.018 (2)	0.033 (2)	0.0006 (15)	0.0045 (16)	−0.0056 (16)
O3	0.030 (2)	0.026 (2)	0.041 (2)	0.0005 (17)	0.0111 (18)	0.0059 (19)
O4	0.032 (2)	0.024 (2)	0.026 (2)	0.0018 (17)	0.0102 (16)	0.0019 (17)
O5	0.033 (2)	0.017 (2)	0.028 (2)	0.0008 (16)	0.0064 (16)	−0.0004 (16)
O6	0.035 (2)	0.020 (2)	0.030 (2)	0.0032 (17)	0.0087 (18)	−0.0037 (17)
O7	0.028 (3)	0.013 (3)	0.032 (3)	0	0.005 (2)	0
O8	0.026 (3)	0.015 (3)	0.036 (3)	0	0.001 (2)	0
F1A	0.085 (3)	0.037 (2)	0.088 (4)	0.018 (2)	0.013 (2)	−0.027 (2)
F2A	0.085 (3)	0.037 (2)	0.088 (4)	0.018 (2)	0.013 (2)	−0.027 (2)
F3A	0.085 (3)	0.037 (2)	0.088 (4)	0.018 (2)	0.013 (2)	−0.027 (2)
F1B	0.085 (3)	0.037 (2)	0.088 (4)	0.018 (2)	0.013 (2)	−0.027 (2)
F2B	0.085 (3)	0.037 (2)	0.088 (4)	0.018 (2)	0.013 (2)	−0.027 (2)
F3B	0.085 (3)	0.037 (2)	0.088 (4)	0.018 (2)	0.013 (2)	−0.027 (2)
F4	0.036 (2)	0.042 (3)	0.090 (3)	0.0088 (19)	−0.012 (2)	−0.025 (2)
F5	0.029 (2)	0.049 (3)	0.070 (3)	0.0060 (17)	0.015 (2)	−0.006 (2)
F6	0.054 (3)	0.040 (3)	0.062 (3)	0.022 (2)	0.005 (2)	0.011 (2)
F7A	0.047 (2)	0.056 (2)	0.091 (3)	−0.0059 (17)	0.0231 (18)	0.031 (2)
F8A	0.047 (2)	0.056 (2)	0.091 (3)	−0.0059 (17)	0.0231 (18)	0.031 (2)
F9A	0.047 (2)	0.056 (2)	0.091 (3)	−0.0059 (17)	0.0231 (18)	0.031 (2)
F7B	0.047 (2)	0.056 (2)	0.091 (3)	−0.0059 (17)	0.0231 (18)	0.031 (2)
F8B	0.047 (2)	0.056 (2)	0.091 (3)	−0.0059 (17)	0.0231 (18)	0.031 (2)
F9B	0.047 (2)	0.056 (2)	0.091 (3)	−0.0059 (17)	0.0231 (18)	0.031 (2)
F10	0.066 (3)	0.052 (3)	0.043 (2)	0.035 (2)	0.007 (2)	−0.003 (2)
F11	0.132 (5)	0.073 (4)	0.094 (4)	0.014 (4)	0.089 (4)	−0.001 (3)
F12	0.067 (3)	0.070 (3)	0.049 (3)	0.018 (3)	−0.003 (2)	−0.029 (2)
F13	0.044 (2)	0.040 (2)	0.043 (2)	0.0144 (18)	−0.0051 (17)	−0.0022 (18)
F14	0.061 (2)	0.032 (2)	0.040 (2)	0.0174 (19)	0.0061 (18)	−0.0043 (18)
F15	0.060 (3)	0.028 (2)	0.107 (4)	0.006 (2)	0.039 (3)	0.019 (2)
F16	0.053 (2)	0.056 (3)	0.033 (2)	−0.014 (2)	0.0104 (18)	−0.0254 (19)
F17	0.038 (2)	0.065 (3)	0.043 (2)	0.0105 (19)	0.0137 (18)	−0.015 (2)
F18	0.073 (3)	0.054 (3)	0.045 (2)	−0.008 (2)	0.034 (2)	−0.001 (2)
C02	0.069 (4)	0.032 (3)	0.061 (5)	0	−0.019 (4)	0
O01	0.069 (4)	0.032 (3)	0.061 (5)	0	−0.019 (4)	0
C01	0.069 (4)	0.032 (3)	0.061 (5)	0	−0.019 (4)	0
C03	0.069 (4)	0.032 (3)	0.061 (5)	0	−0.019 (4)	0

### Geometric parameters (Å, °)

**Table:**

Hf—O1	2.258 (4)	C8—C9	1.368 (9)
Hf—O2	2.147 (4)	C8—H2	0.93
Hf—O3	2.209 (4)	C9—O4	1.262 (7)
Hf—O4	2.150 (4)	C9—C10	1.540 (9)
Hf—O5	2.238 (4)	C10—F10	1.312 (8)
Hf—O6	2.137 (4)	C10—F12	1.323 (9)
Hf—O7	2.113 (3)	C10—F11	1.325 (8)
Hf—O8	2.096 (3)	C11—F14	1.302 (7)
C1—F3B	1.286 (13)	C11—F15	1.329 (7)
C1—F2B	1.288 (13)	C11—F13	1.337 (7)
C1—F3A	1.294 (18)	C11—C12	1.551 (8)
C1—F1A	1.302 (19)	C12—O5	1.239 (7)
C1—F1B	1.389 (14)	C12—C13	1.395 (8)
C1—F2A	1.491 (18)	C13—C14	1.365 (9)
C1—C2	1.514 (10)	C13—H3	0.93
C2—O1	1.238 (7)	C14—O6	1.275 (7)
C2—C3	1.389 (8)	C14—C15	1.523 (8)
C3—C4	1.358 (8)	C15—F16	1.318 (7)
C3—H1	0.93	C15—F17	1.332 (8)
C4—O2	1.269 (7)	C15—F18	1.339 (8)
C4—C5	1.531 (8)	O7—Hf^i^	2.113 (3)
C5—F5	1.309 (9)	O7—H7	0.81 (3)
C5—F6	1.331 (8)	O8—Hf^i^	2.096 (3)
C5—F4	1.340 (7)	O8—H8	0.81 (2)
C6—F7A	1.258 (12)	C02—O01	1.221 (14)
C6—F8B	1.28 (2)	C02—C01	1.496 (14)
C6—F9A	1.311 (11)	C02—C03	1.558 (15)
C6—F9B	1.348 (19)	C01—H01A	0.96
C6—F7B	1.38 (2)	C01—H01B	0.96
C6—F8A	1.419 (14)	C01—H01C	0.96
C6—C7	1.532 (10)	C03—H03A	0.96
C7—O3	1.249 (7)	C03—H03B	0.96
C7—C8	1.394 (10)	C03—H03C	0.96
			
O2—Hf—O1	75.83 (14)	F9B—C6—F8A	129.3 (11)
O4—Hf—O3	74.36 (16)	F7B—C6—F8A	84.5 (11)
O6—Hf—O5	75.77 (14)	F7A—C6—C7	112.2 (8)
O8—Hf—O7	66.82 (18)	F8B—C6—C7	110.8 (10)
O8—Hf—O6	89.34 (11)	F9A—C6—C7	113.9 (8)
O7—Hf—O6	84.20 (11)	F9B—C6—C7	110.9 (11)
O8—Hf—O2	145.72 (16)	F7B—C6—C7	108.5 (9)
O7—Hf—O2	147.46 (15)	F8A—C6—C7	109.4 (7)
O6—Hf—O2	94.45 (16)	O3—C7—C8	126.6 (6)
O8—Hf—O4	110.88 (12)	O3—C7—C6	114.0 (7)
O7—Hf—O4	79.11 (12)	C8—C7—C6	119.4 (6)
O6—Hf—O4	145.55 (15)	C9—C8—C7	117.6 (6)
O2—Hf—O4	84.23 (15)	C9—C8—H2	121.2
O8—Hf—O3	76.26 (14)	C7—C8—H2	121.2
O7—Hf—O3	122.16 (13)	O4—C9—C8	127.9 (6)
O6—Hf—O3	139.27 (16)	O4—C9—C10	111.6 (6)
O2—Hf—O3	78.85 (15)	C8—C9—C10	120.5 (6)
O8—Hf—O5	142.20 (16)	F10—C10—F12	107.5 (7)
O7—Hf—O5	77.11 (16)	F10—C10—F11	107.9 (6)
O2—Hf—O5	71.11 (14)	F12—C10—F11	107.8 (6)
O4—Hf—O5	71.25 (15)	F10—C10—C9	110.8 (5)
O3—Hf—O5	135.93 (14)	F12—C10—C9	111.2 (5)
O8—Hf—O1	73.62 (15)	F11—C10—C9	111.5 (6)
O7—Hf—O1	132.48 (14)	F14—C11—F15	108.8 (5)
O6—Hf—O1	69.78 (15)	F14—C11—F13	108.3 (5)
O4—Hf—O1	141.43 (15)	F15—C11—F13	107.4 (5)
O3—Hf—O1	69.63 (15)	F14—C11—C12	112.5 (5)
O5—Hf—O1	129.42 (15)	F15—C11—C12	110.7 (5)
F3B—C1—F2B	108.8 (9)	F13—C11—C12	109.0 (5)
F2B—C1—F3A	126.8 (10)	O5—C12—C13	127.0 (5)
F3B—C1—F1A	125.3 (10)	O5—C12—C11	114.6 (5)
F3A—C1—F1A	118.0 (12)	C13—C12—C11	118.3 (5)
F3B—C1—F1B	100.6 (9)	C14—C13—C12	119.3 (6)
F2B—C1—F1B	108.3 (10)	C14—C13—H3	120.4
F3A—C1—F1B	64.3 (10)	C12—C13—H3	120.4
F1A—C1—F1B	74.1 (11)	O6—C14—C13	127.4 (5)
F3B—C1—F2A	55.5 (8)	O6—C14—C15	111.8 (5)
F2B—C1—F2A	66.8 (9)	C13—C14—C15	120.8 (6)
F3A—C1—F2A	92.2 (12)	F16—C15—F17	108.1 (5)
F1A—C1—F2A	100.9 (11)	F16—C15—F18	107.2 (5)
F1B—C1—F2A	147.5 (9)	F17—C15—F18	106.9 (5)
F3B—C1—C2	116.0 (9)	F16—C15—C14	110.6 (5)
F2B—C1—C2	115.4 (8)	F17—C15—C14	111.6 (5)
F3A—C1—C2	117.0 (10)	F18—C15—C14	112.2 (5)
F1A—C1—C2	117.6 (10)	C2—O1—Hf	133.0 (4)
F1B—C1—C2	106.4 (8)	C4—O2—Hf	136.3 (4)
F2A—C1—C2	104.2 (9)	C7—O3—Hf	133.8 (4)
O1—C2—C3	127.4 (5)	C9—O4—Hf	134.4 (4)
O1—C2—C1	115.0 (6)	C12—O5—Hf	133.5 (4)
C3—C2—C1	117.5 (6)	C14—O6—Hf	135.3 (4)
C4—C3—C2	120.4 (5)	Hf—O7—Hf^i^	112.5 (3)
C4—C3—H1	119.8	Hf—O7—H7	123.75 (12)
C2—C3—H1	119.8	Hf^i^—O7—H7	123.75 (12)
O2—C4—C3	127.1 (5)	Hf^i^—O8—Hf	113.9 (3)
O2—C4—C5	111.6 (5)	Hf^i^—O8—H8	123.07 (13)
C3—C4—C5	121.2 (5)	Hf—O8—H8	123.07 (13)
F5—C5—F6	108.4 (5)	O01—C02—C01	127.0 (10)
F5—C5—F4	107.1 (6)	O01—C02—C03	118.5 (9)
F6—C5—F4	107.3 (6)	C01—C02—C03	114.5 (13)
F5—C5—C4	111.8 (6)	C02—C01—H01A	109.5
F6—C5—C4	110.5 (5)	C02—C01—H01B	109.5
F4—C5—C4	111.5 (5)	H01A—C01—H01B	109.5
F7A—C6—F8B	123.9 (13)	C02—C01—H01C	109.5
F7A—C6—F9A	113.4 (8)	H01A—C01—H01C	109.5
F8B—C6—F9A	78.5 (9)	H01B—C01—H01C	109.5
F7A—C6—F9B	86.9 (10)	C02—C03—H03A	109.5
F8B—C6—F9B	109.2 (11)	C02—C03—H03B	109.5
F8B—C6—F7B	107.4 (13)	H03A—C03—H03B	109.5
F9A—C6—F7B	131.6 (10)	C02—C03—H03C	109.5
F9B—C6—F7B	110.0 (11)	H03A—C03—H03C	109.5
F7A—C6—F8A	105.0 (9)	H03B—C03—H03C	109.5
F9A—C6—F8A	101.9 (8)		

Symmetry codes: (i) −*x*+1, *y*, −*z*+1/2.

### Hydrogen-bond geometry (Å, °)

**Table:**

*D*—H···*A*	*D*—H	H···*A*	*D*···*A*	*D*—H···*A*
O7—H7···O01	0.81 (3)	1.98 (3)	2.783 (10)	170 (1)
